# Improved Energy Density at High Temperatures of FPE Dielectrics by Extreme Low Loading of CQDs

**DOI:** 10.3390/ma17143625

**Published:** 2024-07-22

**Authors:** Huan Wang, Hang Luo, Yuan Liu, Fan Wang, Bo Peng, Xiaona Li, Deng Hu, Guanghu He, Dou Zhang

**Affiliations:** State Key Laboratory of Powder Metallurgy, Central South University, Changsha 410083, China; 233311011@csu.edu.cn (H.W.); yuanliu1208@csu.edu.cn (Y.L.); 233301056@csu.edu.cn (F.W.); 23311002@csu.edu.cn (B.P.); 223307005@csu.edu.cn (X.L.); 233311054@csu.edu.cn (D.H.); he15773257831@163.com (G.H.); dzhang@csu.edu.cn (D.Z.)

**Keywords:** energy storage density, high-temperature dielectric capacitor, high electron affinity

## Abstract

Electrostatic capacitors, with the advantages of high-power density, fast charging–discharging, and outstanding cyclic stability, have become important energy storage devices for modern power electronics. However, the insulation performance of the dielectrics in capacitors will significantly deteriorate under the conditions of high temperatures and electric fields, resulting in limited capacitive performance. In this paper, we report a method to improve the high-temperature energy storage performance of a polymer dielectric for capacitors by incorporating an extremely low loading of 0.5 wt% carbon quantum dots (CQDs) into a fluorene polyester (FPE) polymer. CQDs possess a high electron affinity energy, enabling them to capture migrating carriers and exhibit a unique Coulomb-blocking effect to scatter electrons, thereby restricting electron migration. As a result, the breakdown strength and energy storage properties of the CQD/FPE nanocomposites are significantly enhanced. For instance, the energy density of 0.5 wt% CQD/FPE nanocomposites at room temperature, with an efficiency (η) exceeding 90%, reached 9.6 J/cm^3^. At the discharge energy density of 0.5 wt%, the CQD/FPE nanocomposites remained at 4.53 J/cm^3^ with an efficiency (η) exceeding 90% at 150 °C, which surpasses lots of reported results.

## 1. Introduction

One of the distinctive features of electrostatic capacitors is their ability to rapidly release stored energy in a very short period of time (on the order of microseconds) to generate powerful power pulses, which makes them play a key role in modern electronic devices and power systems [1,2,3]. Ceramic capacitors are prone to cracking under temperature changes and mechanical stress due to the brittleness of ceramic materials and are large in size [4,5,6]. Film capacitors with polymer dielectrics showing the advantages of high dielectric strength, good processability, and excellent self-healing ability are receiving attention from researchers [7]. However, in extreme environments, especially at high temperatures (more than 150 °C), the energy storage density and efficiency of polymer dielectrics are significantly reduced due to the inevitably increasing conductivity, which cannot meet the demand for electronic devices in emerging applications such as hybrid vehicles, oil and gas exploration, and advanced propulsion systems [8,9]. Currently, commercially available dielectric biaxially oriented polypropylene (BOPP) cannot be operated above 105 °C [10,11]. This is due to the fact that when operating under high electric fields and temperatures exceeding 85 °C, BOPP will result in a significant increase in charge injection and conduction losses, which causes a rapid increase in the leakage current density, leading to a significant decrease in the energy density and storage efficiency [12,13]. Therefore, it is of great significance to develop capacitor dielectric materials that can meet the demands of applications at high temperatures and high electric fields.

Polymers with high $T_{g}$, such as FPE, PEI, PI, PEN, PC, etc., are demonstrated as potential dielectrics that can be applied in high-temperature environments [14,15]. FPE (fluorene polyester) is a class of amorphous poly aryl esters with a $T_{g}$ as high as 330 °C, and a dielectric loss of less than 0.003 over a temperature range of 25~250 °C. It is an ideal choice for high-temperature dielectric materials attracting increasing attention [11,16,17]. The escalation of thermally activated charge transport under high electric fields at elevated temperatures results in an exponential surge in leakage current, thereby precipitating a significant decline in the energy density and efficiency of pure FPE at high temperatures [2]. Therefore, polymer dielectrics with a high glass transition temperature alone are not enough to solve the above problem. The key point for improving the performance of energy storage in extreme application environments lies in how to address the conduction loss of polymer dielectrics under high temperatures and high fields [9]. Studies show that the loss mechanism of polymer dielectrics under high temperatures and high fields is mainly due to the increase in charge injection and carrier migration. Therefore, finding solutions for suppressing carrier injection and transmission is urgent [18]. Among the various methods, constructing nanocomposite dielectrics has been proven to be an effective strategy [19]. Researchers believe that the introduction of wide bandgap inorganic nanofillers, such as BNNS, Al_2_O_3_, MgO, and HfO_2_, into the polymer matrix can create energy traps in composite dielectrics, thereby suppressing conduction loss [7,19,20,21]. Ren et al. [22] prepared nanocomposites featuring fillers with ZrO_2_ cores and Al_2_O_3_ shells into a polyetherimide (PEI) matrix, significantly reducing the leakage current density by an order of magnitude compared to pure PEI. This innovation enabled an energy storage density of 5.19 J/cm^3^ (η > 80%) at 150 °C.

Carbon quantum dots (CQDs) are small, dispersed carbon particles less than 10 nanometers in size, featuring functional groups such as hydroxyl, amino, carboxyl, or aromatic rings on their surface [23,24]. These particles have demonstrated excellent photostability, low cytotoxicity, good biocompatibility, ease of surface modification, and high chemical stability [24,25]. Carbon quantum dots (CQDs) are formed by the random polymerization of linear polymers or precursors that are highly cross-linked and slightly graphitized, combining the properties of both polymers and graphitic carbon [26]. Neutral quantum dots effectively trap electrons, while charged quantum dots can create a potential barrier of e^2^/2C in insulating systems [27]. When this potential barrier significantly exceeds the thermal energy of the electron, it prevents other electrons from passing through, resulting in a Coulomb blockade effect [28,29]. Consequently, uniformly dispersed quantum dots in a polymer matrix can impede carrier migration, leading to enhanced breakdown strength and energy storage capacity. The introduction of carbon quantum dots into a polymer matrix to construct composite dielectric materials exhibits advantages. (1) Compared with inorganic nano-fillers, the rich functional groups on the surface of carbon quantum dots can solve the dispersion and compatibility problems of fillers in polymer matrix, which is conducive to the uniform distribution of electric field; (2) Due to the high electron affinity energy of carbon quantum dots, the carrier transport and migration can be inhibited through the construction of traps; (3) Compared with the semiconductor small molecules with high electron affinity energy, the synthesis process for carbon quantum dots is more simple, economical, and green [30,31,32].

In this study, CQDs with functional groups such as carboxyl and hydroxyl on their surface were synthesized using a simple, mass-producible hydroxyl-acetaldehyde condensation method and incorporated into an FPE matrix for high-temperature capacitive energy storage. The high electron affinity energy of CQDs allows them to capture injected and excited electrons through strong electrostatic attraction, introducing a large trap energy level ($\varphi_{e}$ = 1.54) that makes it difficult for trapped electrons to escape from the trap sites, thereby limiting carrier transport and migration. This reduction in conduction loss significantly enhances the energy storage performance of the composites at high temperatures. For instance, at 150 °C, 0.5 wt% CQD/FPE nanocomposites can achieve a discharge energy density of 4.53 J/cm^3^, with an energy efficiency maintained at 90.4% and a breakdown electric field reaching 596.2 MV/m. 

## 2. Materials and Methods

### 2.1. Materials

The NaOH, acetaldehyde solution (40% in H_2_O), and HCl solution (1 M) were purchased from Sinopharm Chemical Reagent Beijing Co., Beijing, China. FPE particles were supplied by PolyK Technologies, LLC, Philipsburg, PA, USA. The 1-methyl-2-pyrrolidone (NMP) solvent was supplied by Sinopharm and was used without further purification.

### 2.2. Preparation of CQDs

A total of 12 g of sodium hydroxide (NaOH) was mixed with 40 mL of an acetaldehyde solution and stirred for 2 h. Subsequently, dilute hydrochloric acid was added to facilitate dissolution. The mixture was then subjected to centrifugation and washed repeatedly with deionized water until a neutral pH was achieved. The resulting powder was dried at 70 °C for 12 h, yielding approximately 4 (±0.2) g of carbon quantum dot (CQD) powder. The reaction yield was determined to be between 23% and 26%.

Sample purification: Ensuring the purity of CQDs synthesized by this method is paramount. To validate this, we employed the following analytical strategies. Initially, We performed an XPS test on the CQDs powder and detailed results are given later, wherein no Na peaks were discernible in the CQDs. This finding attests to the effectiveness of aqueous washing in removing sodium ion residues. Building on this, we redissolved the CQDs in anhydrous ethanol for additional dialysis and quantified the residual Na content using atomic absorption spectroscopy (AAS). The results of the AAS analysis are summarized in Table 1 below. 

The data indicated that the Na content in the carbon dots was exceedingly low. Furthermore, the Na content in the solution outside the dialysis bag remained virtually unchanged, suggesting that the dialysis method did not notably enhance the purity of the carbon dots. The presence of Na in the external solution may also be attributed to the prolonged use of glassware during the dialysis process. In light of these findings, we conclude that the carbon dots synthesized and purified using our method fully satisfy the requirements of our study.

### 2.3. Preparation of Composites

The preparation of CQD/FPE composites utilized the solution casting method. Initially, the CQDs were ultrasonicated for 15 min to ensure their dispersion in NMP solvent. Subsequently, FPE particles were added to the solution and stirred at 60 °C for 12 h. The resulting mixture was then cast onto a glass plate and placed in a 70 °C blast drying oven for 1 h to partially evaporate the NMP. It was then transferred to a vacuum drying oven at 100 °C and held for 3 h, heated to 150 °C and held for 3 h, heated to 200 °C, and finally left for 18 h to completely remove residual solvents to form a composite film.

### 2.4. Characterizations

The samples were subjected to a comprehensive array of advanced characterization techniques. Transmission electron microscopy (TEM, JEM-1210F, JEOL, Tokyo, Japan) and scanning electron microscopy (SEM, Nova NanoSEM230, FEI, Hillsboro, OR, USA) were utilized to scrutinize the micro-morphology. X-ray photoelectron spectroscopy (XPS, ESCALAB 250xi, Thermo Fisher Scientific, Waltham, MA, USA), X-ray diffraction (XRD, D/max 2550, Rigaku Corporation, Tokyo, Japan), and Fourier Transform Infrared Spectroscopy (FTIR, IS-50, Thermo Fisher Scientific, Waltham, MA, USA) were employed to investigate the structural and compositional attributes. UV–Vis spectroscopy (TU-1901, Shanghai Xipu Instrument Co., Ltd., Liushi, China) provided the raw absorption spectra, from which the band gaps of the carbon quantum dots (CQDs) were determined using the Tauc plot method. The formula in Equation (1) was applied, where α is the absorbance index, h is Planck’s constant, *v* is the frequency, *A* is a constant, *E_g_* is the bandgap energy, and n is 1/2 for direct bandgap semiconductors such as CQDs [3].

(1) $${\left(\alpha hv\right)}^{1/n}=A(hv-E_{g})$$

The mechanical properties were evaluated using an ultra-nanometer hardness tester (UNHT, CSM, Peseux, Switzerland), which yielded load-displacement curves. We calculated the Young’s modulus from these curves employing the Oliver–Pharr method. The indenter is a diamond Berkovich indenter. The geometry of the indenter was a three-sided pyramid, and the loading protocol was as follows: acquisition rate of 10.0 Hz, maximum load of 1.00 mN, loading rate of 6.00 mN/min, unloading rate of 6.00 mN/min, and a pause time of 2.0 s. For electrical property testing, gold electrodes with a sputtered area of 3.14 mm² were deposited on each side of the samples. The dielectric characteristics were assessed with an Agilent 4294A LCR meter over a frequency range of 1 kHz to 10 MHz. The capacitance value (*C_p_*) and loss value (D) were measured, and the dielectric constant (*ε_r_*) was calculated using the formula in Equation (2), where *ε*_0_ is the vacuum dielectric constant (8.85 × 10^−12^ F/m), *S* is the electrode area, and d is the sample thickness [5].

(2) $$\varepsilon_{r}=\frac{C_{p}\times d}{\varepsilon_{0}\times S}$$

Pulsed discharge characterization was conducted with a DCQ-20A discharge measurement system (PolyK Technologies, North Philipsburg, PA, USA). High-temperature dielectric properties were measured using a DMS-500 high-temperature dielectric spectroscopy system, which provided data at 1 kHz over a temperature range of 30 to 220 °C. The displacement hysteresis return line (D-E loop) was analyzed using a TF Analyzer 2000 series (aixACCT, Aachen, Germany) at 10 Hz. The charged energy density (*U*) was derived by integrating the area between the charge curve and the ordinate, while the discharge energy density (*U_e_*) was determined by the area between the discharge curve and the ordinate. The efficiency (*η*) was calculated as the ratio of U_e_ to U [33]. Cyclic voltammetry curves were obtained using a CHI electrochemical workstation, and the lowest unoccupied molecular orbital (LUMO) and highest occupied molecular orbital (HOMO) energies were calculated from the reduction potential (*E_red_*) using the Equations (3) and (4) [34].

(3) $$E_{LUMO}=-e(E_{red}+4.40)$$

(4) $$E_{HOMO}=E_{LUMO}-E_{g}$$

The spatial distribution of the electrostatic potential for the FPE model was elucidated through Density Functional Theory (DFT) computations grounded in first-principles calculations. The wavefunction of the molecule under investigation was derived from the solution of the fundamental Schrödinger equation. In this study, DFT calculations were conducted using the B3LYP hybrid functional and the 6-311G(d) basis set without imposing restrictive symmetry constraints within the Gaussian 16 computational package.

## 3. Results 

**Figure 1:**
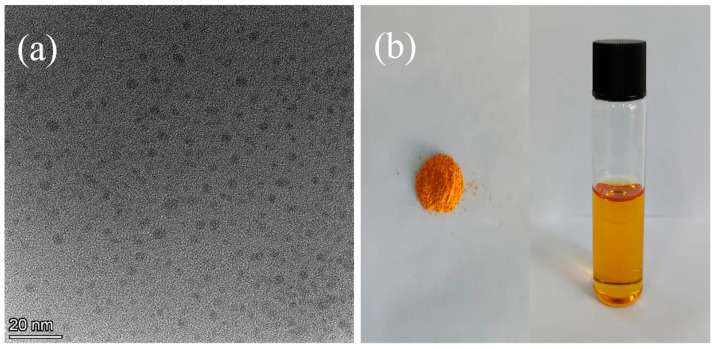
(**a**) TEM images of CQDs under different magnifications, and (**b**) photographs of CQD powder and solution in NMP solvent.

Figure 1a presents TEM images obtained from the CQD powder at various magnifications. Figure 1b displays photographs of the yellow CQD powder and its solution in NMP, revealing a homogeneous and transparent state, indicative of excellent dispersion in the NMP solvent.

**Figure 2:**
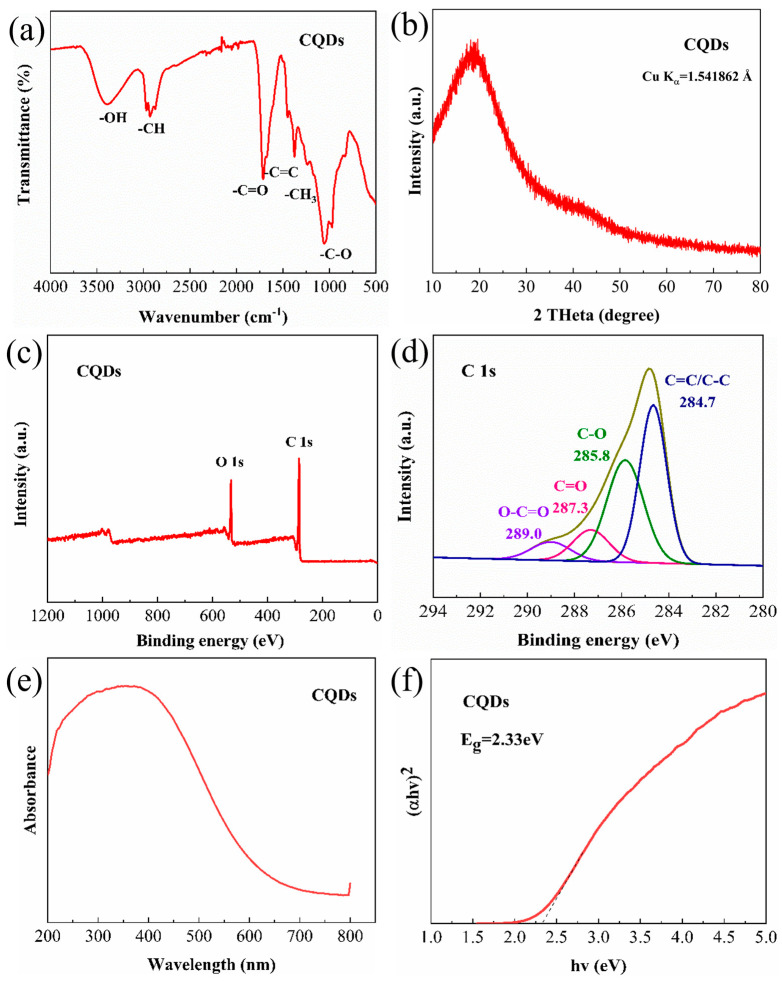
Characterization data of the carbon quantum dots. (**a**) FTIR spectrum, (**b**) XRD pattern, (**c**) full XPS spectrum, (**d**) high-resolution C1s XPS spectrum, (**e**) UV–Vis absorbance spectroscopy, and (**f**) (*αhv*)^2^ − *hv* plot, which is a common method for determining the bandgap energy of semiconductors.

Figure 2a depicts the FTIR spectrum of the CQDs, highlighting absorption bands at 3424 cm^−1^ (associated with -OH stretching), 2971 cm^−1^ (-CH stretching), 1716 cm^−1^ (-C=O stretching), 1681 cm^−1^ (-C=C stretching), 1378 cm^−1^ (-CH_3_ stretching), and 1073 cm^−1^ (-CO stretching). XPS plots (Figure 2c,d) confirmed the presence of two types of carbon: graphitized carbon (C=C and C-C) with binding energy at 284.7 eV and aliphatic carbon (C-O, C=O, and O=C-OH) with binding energies at 285.8 eV, 287.3 eV, and 289.0 eV. Both FTIR and XPS analyses suggest the presence of oxygen-containing functional groups, such as hydroxyl and carboxyl, which contribute to the good dispersion and interfacial compatibility of the CQDs within the polymer matrix. The XRD pattern (Figure 2b) shows a single broad diffraction peak centered around 2θ = 18°, indicative of a highly disordered carbon structure consistent with the TEM observations. The UV–visible absorption spectra (Figure 2e) were used to calculate the ${\left(\alpha h\nu \right)}^{2}-h\nu$ curve (Figure 2f) using the equation ${\left(\alpha h\nu \right)}^{2}=A(h\nu -E_{g})$, where *α* is the absorption coefficient, *h* is Planck’s constant, *ν* is the optical frequency, and *E_g_* is the band gap energy [35]. This analysis determined a band gap ($E_{g}$) of approximately 2.33 eV for the CQDs, confirming their semiconductor nature. 

**Figure 3:**
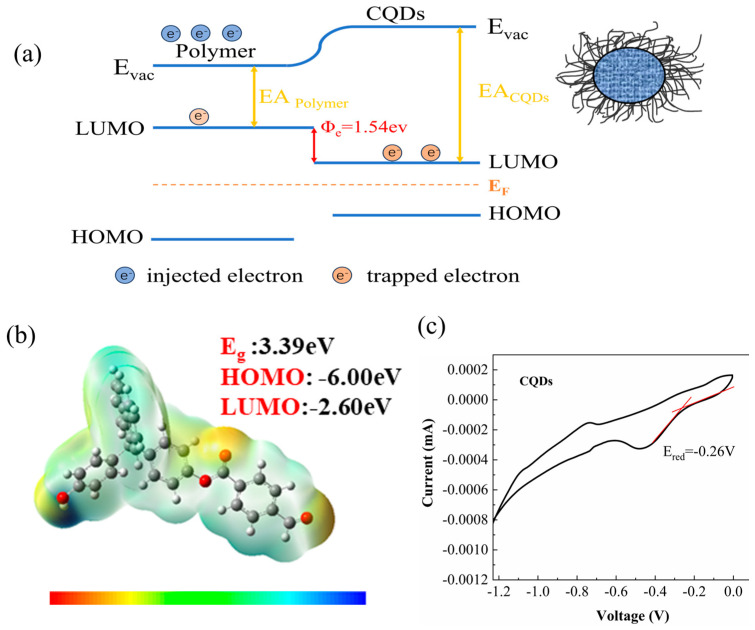
(**a**) Band diagram illustrating the interface between the CQDs filler and the FPE matrix, (**b**) electrostatic potential diagrams and corresponding energy band values for FPE, (**c**) cyclic voltammetry (CV) curves of the CQDs, revealing their electrochemical properties.

The efficacy of CQDs in mitigating charge accumulation within dielectrics and curbing conductivity losses stems from their elevated electron affinity energy. This property enables the formation of a trap energy level $\left(\varphi_{e}={EA}_{CQDs}-{EA}_{polymer}\right)$ within the polymer matrix, facilitating the capture of substantial charge injected by metal electrodes [35,36]. Electrostatic potential maps and energy band values of the FPE, as derived from density functional theory (DFT) simulations, are depicted in Figure 3b. The lowest unoccupied molecular orbital (LUMO) energy level of the FPE was determined to be −2.60 eV. To ascertain the electron affinity energies of the carbon quantum dots (CQDs), cyclic voltammetry tests were conducted using a conventional three-electrode electrochemical testing setup, yielding the cyclic voltammetry curves presented in Figure 3c. The onset reduction potential ($E_{red}$) of the polymer dot, discernible from the figure, was −0.98 eV. The bandgap of CQDs was calculated by Equations (3) and (4); the LUMO energy level of the polymer was calculated to be −4.14 eV. Considering the definition of electron affinity energy (the energy necessary for electrons to reach the vacuum energy level from the bottom of the conduction band) the value is the energy difference from the conduction band’s base to the vacuum level (*E_vac_*) [9]. Hence, EA_CQDs_ = E_vac_ − E_LUMO_ = 4.14 eV [35]. The integration of CQDs thus facilitates the establishment of a profound trap energy level, as illustrated in Figure 3a, with a depth of 1.54 eV.

**Figure 4:**
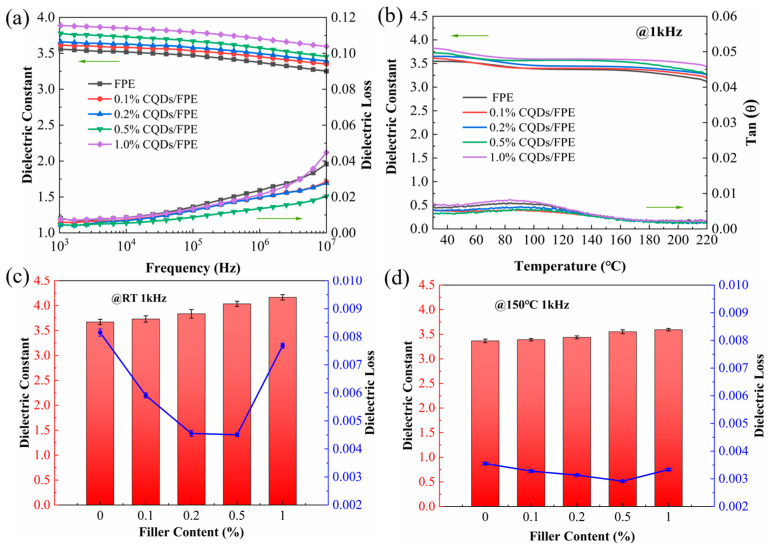
(**a**) Frequency dependence and (**b**) temperature dependence of dielectric constant and dielectric loss of nanocomposites. Changing law of dielectric constant and dielectric loss of nanocomposites with filler content at room temperature (**c**) and 150 °C (**d**). The green arrow serves to denote the coordinate axis pertinent to the data, thereby enhancing the clarity of the visual representation.

The variation of dielectric constants and loss in the composites with frequency is depicted in Figure 4a. The increase in the dielectric constant correlated with the rising content of filler CQDs, attributed to the expansion of the interfacial region within the composites, which enhances interfacial polarization due to the incorporation of CQDs [37]. The dielectric loss initially decreased and then increased as the CQD content rose. Notably, the dielectric loss for all composites was lower than that of pure FPE (0.008) at 1 kHz, primarily due to the Coulomb-blocking effect of CQDs [28,29]. Neutral CQDs act as traps in the electron transport pathway, while charged CQDs create an energy barrier impeding electron flow through the polymer matrix, thereby reducing conductive loss. At lower filler concentrations, the Coulomb-blocking effect intensifies with increasing filler content, impacting the dielectric loss more significantly than interfacial polarization. However, once the filler content exceeds a certain value, the reduced spacing between CQDs facilitates electron conduction, and the inhibition of electron migration diminishes, resulting in an increase in conductive loss [38]. Consequently, samples with 1.0 wt% CQDs exhibited higher dielectric losses than those with 0.5 wt% CQDs. The dielectric constant and loss of all thin film samples remained stable across the frequency range from 10^2^ Hz to 10^4^ Hz, with the dielectric loss beginning to rise only after the frequency exceeds 10^4^ Hz.

Figure 4b illustrates the variation of dielectric properties with the temperature at 1 kHz, and the dielectric loss remained below 0.01 across the temperature range from 30 °C to 220 °C, a characteristic attributed to the high glass transition temperature of FPE [39]. Notably, the dielectric loss decreased with increasing temperatures at 120 °C, a trend that is advantageous to achieve a superior high-temperature energy storage performance. To facilitate a clearer comparison of the dielectric properties among all samples, the values of dielectric constant and loss measured at room temperature and 150 °C for pure FPE and composite dielectrics at 1 kHz are presented in Figure 4c,d. At 150 °C, the dielectric constant of the 0.5 wt% CQD/FPE composite increased from 3.36 to 3.56 compared to pure FPE, and the dielectric loss decreased by 18.8% at high temperatures relative to that of pure FPE.

To examine the impact of CQDs on the breakdown strength of the polymers, the breakdown field strength and stability of the nanocomposites were analyzed using the Weibull distribution [20].

(5) $$P(E)=1-exp{\left(-\left(\frac{E}{E_{b}}\right)\right)}^{\beta }$$

**Figure 5:**
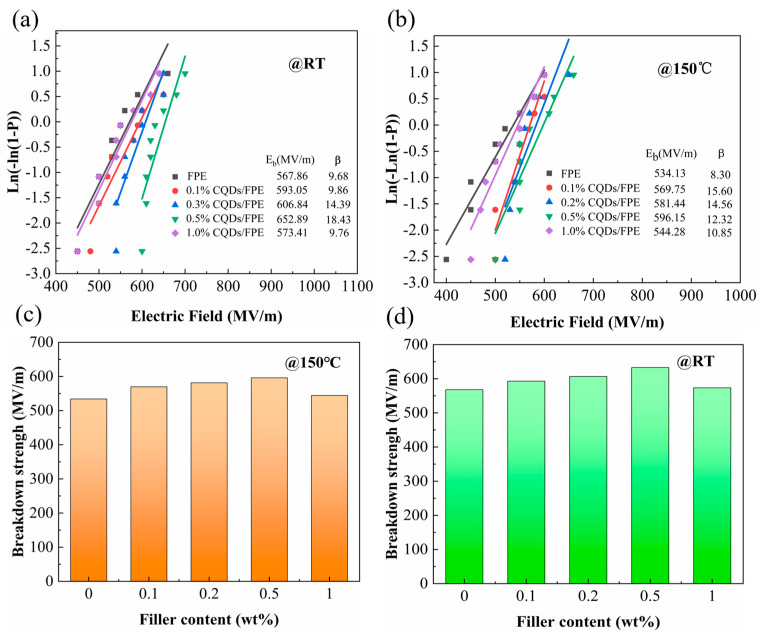
Weibull distribution of breakdown electric field at RT (**a**) and 150 °C (**b**), and characteristic breakdown electric field strength at RT (**c**) and 150 °C (**d**).

The text discusses the cumulative failure probability, *P*(*E*), where *E* represents the tested breakdown strength value, *β* is a shape parameter reflecting the sample data dispersion, and *E_b_* is the characteristic breakdown strength for a specimen with a 63.2% breakdown probability [40]. Figure 5a,b illustrates the Weibull distribution of nanocomposites with varying filler contents at room temperature and 150 °C. To better depict the temperature-dependent variation in the breakdown field strength of the composites, Figure 5c,d presents a bar graph summarizing the trend of breakdown field strength with filler content. It is evident that the breakdown strength of the nanocomposites initially increased and then decreased as the CQDs content rose. Notably, the breakdown strength peaked at 632.8 MV/m for a 0.5 wt% CQD content, a substantial improvement over the pure FPE’s 567.86 MV/m. At 150 °C, the 0.5 wt% CQD/FPE exhibited a breakdown strength of 596.15 MV/m, which was 11% higher than that of the pure FPE. This trend is attributed to the small number of CQDs enhancing the breakdown strength due to their high electron affinity energy and distinctive Coulomb-blocking effect [41]. However, increasing the filler content beyond this point resulted in reduced inter-filler distances, which can form conductive paths and diminish the material’s breakdown strength [42].

**Figure 6:**
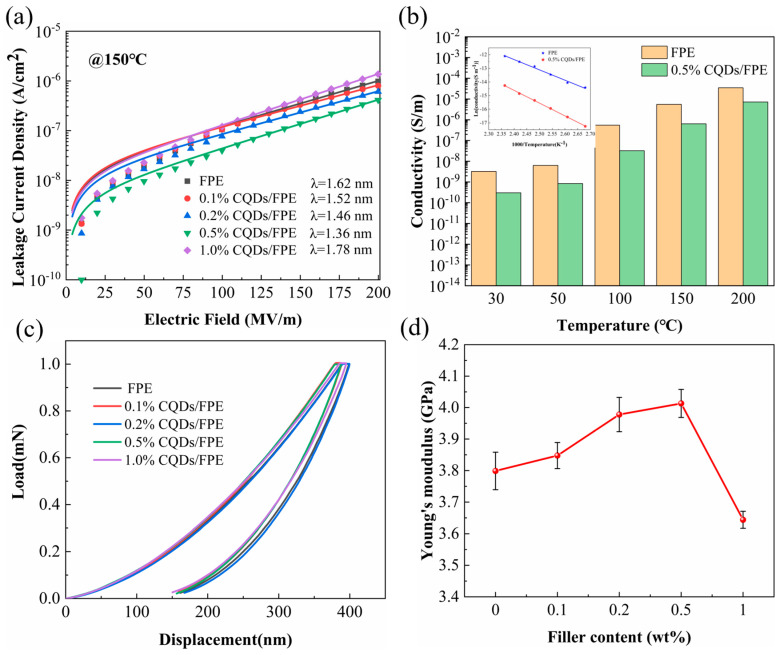
(**a**) Leakage current density at an electric field strength of 200MV/m and (**b**) conductivity variation with temperature of composites. (**c**) Load-displacement curve and (**d**) Young’s modulus as a function of filler content of the nanocomposites.

Figure 6a,b displays the results of leakage current density and conductivity tests for pure polymer and composite dielectrics at 150 °C. The introduction of CQDs significantly reduced these values by an order of magnitude. For instance, the leakage current density and conductivity of the 0.5 wt% CQD/FPE dropped from 1.02 × 10^−6^ A cm^−2^ and 5.52 × 10^−6^ S m^−1^ for the FPE to 4.26 × 10^−7^ A cm^−2^ and 6.40 × 10^−7^ S m^−1^, respectively. In addition, we found that the leakage current density of all nanocomposites increased exponentially with an increasing electric field, indicating that the main charge transfer mechanism in the materials was hopping conduction. We derived the jump distance (average spacing between trap points) by fitting the J-E data to the jump conduction model, utilizing the expression for jump conduction:

(6) $$J=n_{c}v\lambda e\times \exp\left(\frac{W_{a}}{K_{B}T}\right)\sinh(\frac{\lambda eE}{2K_{b}T})$$

Here, *n_c_* denotes the carrier concentration, *λ* represents the hopping distance, *v* signifies the attempt-to-escape frequency, *Wa* is the activation energy expressed in electron volts (eV), *e* is the elementary charge of the carriers, *T* is the temperature, and *K_b_* is the Boltzmann constant [28]. The 0.5% CQD/FPE, exhibiting the smallest λ (1.36 nm), demonstrated the highest trap density compared to the composite film with the pure FPE and other composites containing varying filler concentrations, demonstrating the largest trap density, thereby enhancing the *E_b_* [29]. The plot of ln(J) versus 1/T is provided, illustrating the temperature dependence of the current. The activation energies determined for the pure FPE and the composites with 0.5% CQDs were 0.65 eV and 0.81 eV, respectively. This indicates that CQDs with higher electron affinity energies can establish trap energy levels within the polymers, effectively trapping and binding charges [9]. The breakdown mechanisms of polymer dielectrics encompass mechanical breakdown alongside electrical and thermal breakdown. When the applied stress surpasses the yield strength of the nanocomposite film, the gold electrodes, formed through sputtering, will fail and initiate a discharge, ultimately leading to the film’s breakdown [20,43]. To delve deeper into the impact of the material’s mechanical strength on the breakdown strength, nanoindentation tests were conducted on the nanocomposite film, yielding load-displacement curves and Young’s modulus values, as shown in Figure 6c,d. As the CQDs filler content increased, the Young’s modulus of the composites rose. Due to the abundant functional groups on the CQDs surface, CQDs could form a strong bonding network with the FPE matrix. This leads to enhanced interfacial compatibility between the filler and the FPE matrix, resulting in a more uniform filler distribution and an increased Young’s modulus for the nanocomposites. However, as the filler content continues to increase, partial agglomeration of CQDs may occur, leading to uneven forces within the nanocomposite, thus causing Young’s modulus to initially increase and then decrease with rising filler content [44].

## 4. Discussion

**Figure 7:**
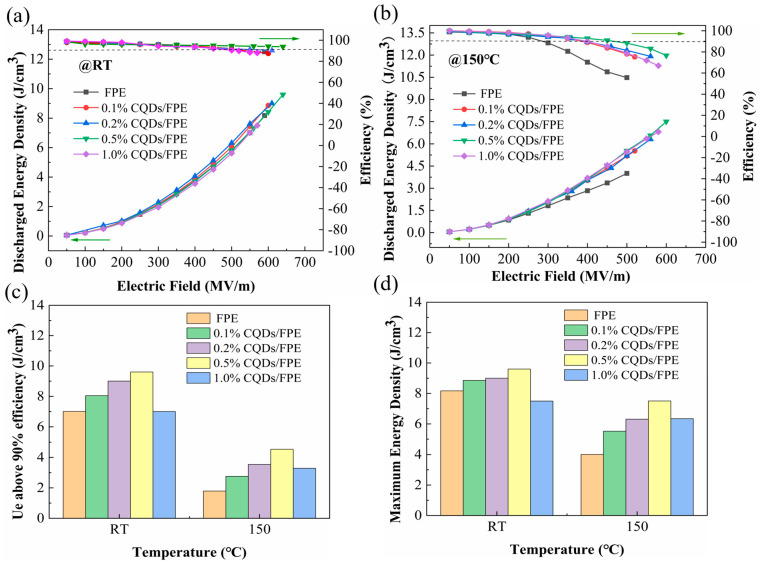
The energy density and efficiency of pure FPE and CQD/FPE nanocomposites at (**a**) RT and (**b**) 150 °C. (**c**) The energy density above 90% efficiency, and (**d**) the maximum energy density. The green arrow serves to denote the coordinate axis pertinent to the data, thereby enhancing the clarity of the visual representation.

The energy storage density and efficiency of the composites at room temperature and 150 °C are depicted in Figure 7a,b. Notably, the sample with 0.5 wt% CQDs achieved the highest breakdown strength of 632 MV/m and an energy density of 9.6 J/cm^3^ (η > 90%). It was also significant that both the pure FPE and its composite dielectric maintained efficiencies above 88%. At 150 °C, the 0.5 wt% CQD/FPE composite reached an efficiency of 90.34% and an energy storage density of 4.53 J/cm^3^, which was 2.53 times greater than the 1.79 J/cm^3^ of pure FPE. Figure 7c illustrates the relationship between composite energy storage density and filler content at room temperature and 150 °C, with efficiencies exceeding 90%. Figure 7d similarly shows the trend of maximum energy storage density with filler content at both temperatures. It is evident that the energy storage density and the maximum energy storage density for composites with efficiencies above 90% exhibited a trend of increasing and then decreasing as filler content increases.

**Figure 8:**
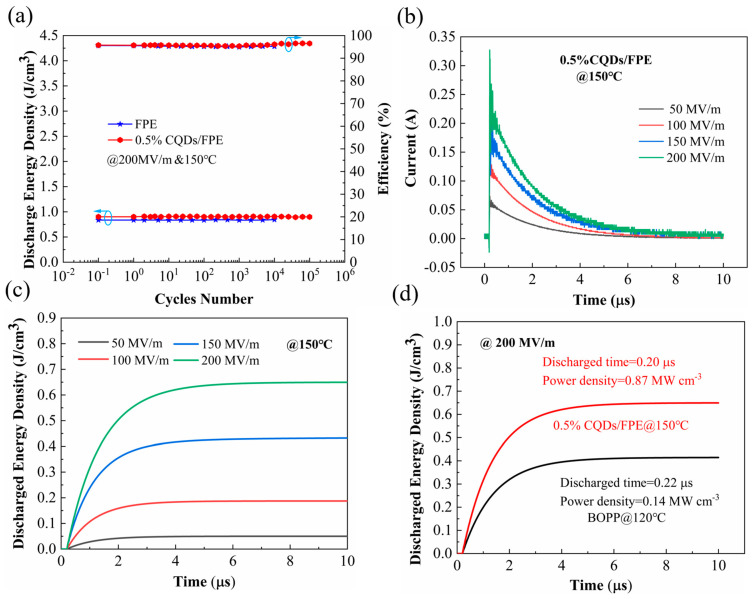
(**a**) Charge–discharge cycle performance of pure FPE and composites. (**b**) Overdamped discharge curves and (**c**) time dependence of W_d_ under different electric fields of the composites with 0.5 wt% CQDs. (**d**) Time dependence of W_d_ under an electric field of 200 MV/m 200 MV/m of commercial BOPP and the composites with 0.5 wt% CQDs. The blue circle serves to denote the coordinate axis pertinent to the data, thereby enhancing the clarity of the visual representation.

Furthermore, Figure 8a presents the $U_{e}$ and *η* results after 10^5^ charge/discharge cycles at 150 °C, 200 MV/m, and 1 kHz. The cycling test revealed no degradation in sample performance, with a slight improvement, indicating excellent cycling stability. The 0.5 wt% CQD/FPE nanocomposites outperformed pure FPE in this regard. The actual pulse discharge energy densities were assessed using a 10 kΩ load resistor to create an overdamped state circuit at 150 °C. The 0.5 wt% CQD/FPE nanocomposites underwent cycling tests at electric fields ranging from 50 to 200 MV/m and at 1 kHz. The $U_{e}$ and *η* results post-105 cycles are shown in Figure 8a. Figure 8b displays the overdamped discharge curves of FPE across the 50 to 200 MV/m electric field range. The pulsed discharge energy density (*W_d_*) was calculated using the formula in Equation (7):

(7) $$W_{d}=R\int I^{2}\left(t\right)dt/V$$

where *R*, *I*, *t*, and *V* represent load resistance, current, time, and sampling volume, respectively [45]. The calculated curves are presented in Figure 8c,d, with a high *W_d_* of 0.61 J/cm^3^ achieved at 150 °C and an electric field of 200 MV/m, along with a rapid discharge time of 10 μs, surpassing the 0.43 J/cm^3^ (120 °C) of commercial BOPP films.

Table 2 compares the energy storage properties of 0.5 wt% CQD/FPE composites with those of several polymer matrix composites reported in the literature. At 150 °C, the performance of the composites in this study surpassed that of most reported dielectric materials. The 0.5 wt% CQD/FPE composites exhibited a synergistic enhancement in both the dielectric constant and breakdown strength while maintaining a high energy efficiency.

## 5. Conclusions

In summary, CQDs were synthesized via a hydroxyl aldehyde condensation method. Due to the abundant surface functional groups, CQDs enable uniform dispersion in the polymer matrix. They also exhibit high electron affinity energy and a unique Coulomb-blocking effect, which can trap migrating carriers and reduce their migration rate. The results indicated that the addition of CQDs significantly improved the dielectric constant and breakdown strength of the composites. At room temperature, the breakdown strength of 0.5 wt% CQD/FPE composites reached 632.89 MV/m, a 13% increase over that of the pure polymer. The energy storage density of the composites was 37% greater than that of pure FPE, and the energy efficiency exceeded 90%. At 150 °C, the incorporation of CQDs effectively mitigated the rapid decline in breakdown strength, energy density, and energy efficiency typically observed in pure FPE as the temperature increased. The 0.5 wt% CQD/FPE composites demonstrated a breakdown strength of 596.15 MV/m, an 11% improvement over the pure polymer, and an energy storage density of 4.53 J/cm^3^, which represents a 2.53-fold increase compared to that of pure FPE. 

## Figures and Tables

**Table 1:** Na Content Analysis: Sodium Ion Concentration in Pre- and Post-Dialysis Solutions and External Dialysis Bag Solution.

Samples	Dialysis Time (h)	Na Content (μg/mL)
Undialyzed stock solution	0	1.056
Solution outside dialysis bag	9	0.474
Solution outside dialysis bag	18	0.535
Solution outside dialysis bag	30	0.506
Solution outside dialysis bag	36	0.501
Dialyzed 36 h stock solution	36	1.034

**Table 2:** Comparison of energy storage properties with the other composites at 150 °C [3,11,27,46,47,48,49,50].

Matrix	Filler	E_b_ (MV/m)	U_e_ (J/cm^3^)	η (%)	References
PEI	BNNS@ST	460	4.29	>80	Compos. Pt. A-Appl. Sci. Manuf. 2023, 175, 9, 107791 [11]
PC	Au	518	4.84	>90	Mater. Chem. A 2022, 10, 18773 [27]
PEI	ZIF 8–67	564	2.96	>90	Small 2023, 19, 12 [3]
PEI	BNNPs	580	4.2	>90	Mater. Chem. C 2022, 10, 13157 [46]
PEI	BN/BaTiO_3_	507	5.23	>90	Sci. China-Mater. 2023, 66, 2652 [47]
PEI	PLZST@Al_2_O_3_	489	10.2	83.5	Mater. Chem. A 2023, 11, 7227 [48]
PI	MgO	450	2.6	89	Polymers 2022, 14, 10, 2918 [49]
PI	BNNS	489	2.58	>90	Adv. Compos. Hybrid Mater. 2022, 5, 238 [50]
FPE	CQDs	596	4.53	>90	This Work

## Data Availability

Data are contained within the article.
